# Moral Stories Can Promote Honesty in Chinese Young Children

**DOI:** 10.3390/bs15060733

**Published:** 2025-05-25

**Authors:** Yanyan Sai, Mo Zheng, Yeqing Tang, Liyang Sai, Xue Liu

**Affiliations:** 1 Mental Health and Counseling Center, Zhejiang University of Science and Technology, Hangzhou 310023, China; saiyanyan@zust.edu.cn; 2 Department of Psychology, Hangzhou Normal University, Hangzhou 311121, China; zhengmo@stu.hznu.edu.cn (M.Z.); tyq_sherlock1372@163.com (Y.T.); 3 Department of Education, Zhejiang Normal University, Jinhua 321004, China

**Keywords:** moral story, children, honesty, lying

## Abstract

Stories are widely used by parents or educators to teach children the virtue of honesty. However, the existing empirical findings on the effect of story-telling on children’s honesty are limited and mixed. This study examined whether moral stories involving honesty can promote honesty in Chinese preschool children (N = 208). The Temptation Resistance Paradigm (TRP) was used to assess children’s honesty. Study 1 showed that children in the positive moral story condition were more likely to tell the truth than those in the control condition, while negative moral story-telling did not have this effect. Study 2 further examined whether combining external appeals with positive moral story-telling could further promote children’s honesty, and the results showed that the combination of the two techniques was equally as effective as moral story-telling alone. These findings have important implications for moral development and moral education.

## 1. Introduction

Honesty is a fundamental virtue across various societies and cultures. Dishonest behavior can erode the interpersonal and institutional trust that is essential for healthy relationships and well-functioning communities. Consequently, parents and educators have made efforts to instill honesty in children from an early age. One widely-used approach is telling children moral stories that emphasize the value of honesty, allowing them to learn this virtue from the stories. However, the empirical evidence regarding the effectiveness of this approach remains limited and controversial. Building on previous research, the present study examined whether story-telling can promote honesty in a sample of Chinese preschool children, and whether combining story-telling with other honesty promotion techniques, such as external appeals, could more effectively promote children’s honesty compared to story-telling alone.

Children start to tell lies as early as 2.5 years old, and as their cognitive capacities develop—specifically, their executive function and theory of mind—they tend to tell more lies (Evans & Lee, 2013). For example, research has shown that approximately 35% of 3-year-old children lie to conceal their rule-violation in the Temptation Resistance Paradigm, and by the age of 5-years-old, around 90% of 5-year-old children lie to conceal their rule-violation (Talwar & Lee, 2008). During late childhood and adolescence, high rates of lying have been found to be associated with various behavioral problems, such as disruptive and aggressive behaviors (e.g., Gervais et al., 2000; Zanette et al., 2020). Therefore, it is important to develop techniques to promote children’s honesty. Researchers have identified several techniques to promote children’s honesty. For example, these techniques include having children make an honest promise (Evans & Lee, 2010; Heyman et al., 2015; Kanngiesser et al., 2021), enhancing their self-awareness (Beaman et al., 1979; Bender et al., 2018; Ding et al., 2019), and telling them moral stories (Butean et al., 2021; Ding et al., 2023, 2024; Lee et al., 2014; Talwar et al., 2016, 2018). Story-telling is one promising approach to promoting honesty in children, because moral stories not only convey important moral lessons, but also easily capture children’s interest (Lee et al., 2014). In addition, the main theoretical basis for story-telling is the social learning theory, which suggests that people can learn social norms, such as honesty, by observing others, even if they are merely characters in a story (Bandura, 2001; Ding et al., 2024). While the theoretical foundation is sound, existing empirical findings on the effect of story-telling on children’s honesty are mixed. In the first empirical study examining this question (Lee et al., 2014), children aged 3 to 7 years in Canada were asked not to peek at a toy when the experimenter left during the Temptation Resistance Paradigm. Given the high temptation of the toy, most of the children peeked. When the experimenter returned, they told one of several different types of moral stories and then asked children whether they had peeked. Results showed that children who heard a moral story emphasizing truth-telling with positive consequences (called a positive moral story) were more likely to confess their transgression than children who heard a neutral story unrelated to honesty, but there was no significant difference in children’s confession rates between hearing a moral story emphasizing lie-telling with negative consequences (called negative moral story) and hearing a neutral story unrelated to honesty. These results indicate that moral stories emphasizing confession with positive consequences can promote children’s honesty. This finding was subsequently replicated by two studies, which found that moral stories emphasizing confession with positive consequences made children in Canada more likely to tell the truth about others’ transgressions (Talwar et al., 2016, 2018). However, some other studies have found that moral stories do not promote children’s honesty. For example, using a similar temptation resistance paradigm, researchers found that neither moral stories with positive consequences nor those with negative consequences led to increased confessions from children aged 3 to 6 in Romania (Butean et al., 2021). Similarly, a recent study showed that moral stories emphasizing positive consequences for honesty did not promote honesty among children aged 5 to 6 in Singapore, although an effect was observed in younger children aged 3 to 4 (Ding et al., 2023). Furthermore, positive moral stories were found to be effective only when children were encouraged to try being like the protagonist in the story (Ding et al., 2024).

Given the limited and mixed evidence regarding the effect of moral stories on children’s honesty, it is crucial to conduct more studies to investigate this influence. Such research can better inform parents and educators on how to effectively use moral stories to teach children honesty. Thus, the first aim of the present study is to examine whether moral stories can promote children’s honesty by using a sample of Chinese children. Moreover, while previous studies found that positive moral stories promoted children’s honesty, the effect was relatively weak. For example, in the study of Lee et al. (2014), the percentage of children who told the truth in the positive moral story (50%) was only twenty percent more than that in the control condition (around 30%); half of children still lied to the experimenters after positive moral story-telling. Thus, it is important to find techniques to further promote children’s honesty. One possible approach is combining moral story-telling with other honesty-promotion techniques (e.g., Evans & Talwar, 2024). External appeals that emphasize telling the truth to please adults seem to be a good candidate to be combined with moral story-telling. Children’s behavior is largely influenced by how adults react to what they do, such as being approved of by parents or trying to please adults (Talwar et al., 2015). For example, Talwar et al. (2015) found that external appeals emphasizing that the experimenter will be happy if the children tell the truth make children more likely to admit to their rule-violation compared to a control condition. Thus, the second aim of this study (Study 2) was to examine whether expressing that the experimenter will be happy if children respond honestly after telling moral stories would lead to more confessions than simply telling a moral story alone.

To address the aforementioned issues, Study 1 recruited 5- to 6-year-old children to participate in a modified Temptation Resistance Paradigm (TRP), which is widely used in research on children’s honesty (Lewis et al., 1989; Talwar & Lee, 2002). In this task, the child was left alone in a room, being told not to turn around to peek at a toy. Upon the experimenter returning to the room, she told the child a moral story, and then asked the child whether he/she had peeked at the toy. Children were randomly assigned to three story conditions: the positive moral story condition, the negative moral story condition, and the neutral story condition. Consistent with previous research, the positive moral story emphasizes that the story character tells the truth with positive consequences, the negative moral story emphasizes that the story character tells a lie with negative consequences, and the neutral story is one that is not related to honesty. However, unlike previous research, we used moral stories that were developed from traditional Chinese stories in which the characters and contexts differ from those in earlier studies (see Section 2.1 for details). This aims to examine whether the effect of moral stories on children’s honesty holds across different story contexts. We included only 5- to 6-year-old children because our pilot study showed that many 3-year-old children did not understand the stories we told. Additionally, previous studies found that age did not influence the effect of moral stories on children’s honesty (Lee et al., 2014; Talwar et al., 2016, 2018).

Based on previous findings, we hypothesized that more children would confess to peeking in the positive moral story condition compared to the neutral story condition, but that there would be no significant difference in confession rates between the negative moral story condition and the neutral story condition (Ding et al., 2024; Lee et al., 2014; Talwar et al., 2016).

## 2. Study 1

### 2.1. Method

#### 2.1.1. Participants

A total of 112 children of the Han nationality were recruited from a kindergarten in an eastern city in China. Participants were randomly assigned to three conditions: the control condition, the positive moral story condition, and the negative moral story condition. Four children were excluded because they did not understand the story. The valid participants in each condition were: 36 children in the control condition (*M*_age_ = 5.85 years, *SD* = 0.39, range = 5.22 to 6.58, 16 boys), 36 children in the negative moral story condition (*M*_age_ = 5.76, *SD* = 0.44, range = 5.21 to 6.81, 22 boys), or 36 children in the positive moral story condition (*M*_age_ = 5.77, *SD* = 0.31, range = 5.22 to 6.18, 20 boys). The sample size was determined by a prior power analysis using G*Power 3.1 (Faul et al., 2009) with the Power (1–*β*) set at 0.80, *α* = 0.05, and the effect size (odds ratio) = 3.1. The effect size used was based on the study of Lee et al. (2014). The results showed that, to detect a significant condition effect in a binary logistic regression analysis, 97 children with peeking behavior (around 33 children per condition) were required. So, under each condition, we stopped recruiting participants once 36 children had peeked. The study received informed consent from parents or legal guardians, and children provided verbal assent before participating. All studies were approved by the Ethical Review Committee of Hangzhou Normal University.

#### 2.1.2. Procedure

The experiment employed a modified Temptation Resistance Paradigm, was carried out in a quiet room in the kindergarten, and each child was tested individually. At the beginning of the experiment, the experimenter (female) told the child to play a game of guessing the toy by listening to the sound. The game had three trials, and if the toys were correctly guessed three times, a prize would be awarded. After the game started, the experimenter asked the child to turn back to the table and guess what the toy on the table was based on the sound they heard. The first two toys were directly related to the sound (such as a “meow” corresponding to a toy cat and a “woof” corresponding to a toy dog), and the child could easily guess the toy. Before the third toy was guessed, the experimenter made an excuse to leave the room. Before leaving, she told the child: “I’m going to read you a story later, now go downstairs and get the storybook, and don’t look back at the toy when I left”. Then, the experimenter placed a new toy on the table (the toy’s sound did not match the toy, such as playing an unrelated piece of music, and the child was unlikely to guess the name of the toy based on its sound). A hidden camera was placed in the room to record whether the child peeked.

One minute later, the experimenter returned to the room and quickly covered the toy with a cloth. Then, the experimenter told the child a story based on the condition the child assigned: positive moral story, negative moral story, or control condition. All stories were roughly the same length. The children in the control condition were told a story about a character named Zu Ti, who gets up early every morning to practice swordsmanship. This story is not related to honesty. The children in the negative moral story condition heard a story about a character named Sima Guang, who told his father that he had found a way to make walnuts easier to peel. After his father discovered the truth, he punished Sima Guang for lying. The children in the positive moral story condition heard a story about a character named Guo Moruo, who confessed to picking flowers from his uncle, and his uncle praised him for his honesty. After finishing reading the story, to ensure that the child could understand the story, the experimenter asked one check question (for example, “Why was Sima Guang punished by his father?”). After the child answered the story comprehension questions, the experimenter began to ask the two target questions: “Did you turn around and peek at the toy when I left the room?” Then she asked, “And guesses what the third toy is?” The experimenter recorded the child’s responses and behavioral responses. The number of children who confessed was used as the main dependent variable in this study.

### 2.2. Results

**Figure 1:**
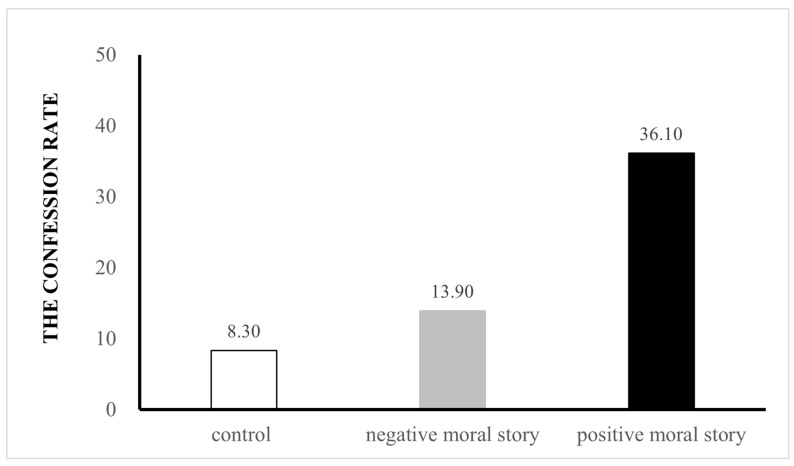
Percentage of children who confessed in Study 1.

Preliminary analyses revealed that child’s gender was not significantly related to lying behavior, χ^2^ (1) = 1.23, *p* = 0.27, and that age was not significantly related to lying behavior (Spearman *r* = 0.07, *p* = 0.49). Thus, gender and age were removed as factors for all further analyses. All the children peeked at the toy after the experimenter left. Of the children who peeked at the toy, only 19.4% of children (N = 21) confessed their transgressions: 8.3% (N = 3) of children in the control condition confessed, 13.9% (N = 5) of children in the negative moral story condition confessed, and 36.1% (N = 13) of children in the positive moral story condition confessed (see Figure 1). To examine the effect of story-telling on children’s confession behavior, a binary logistic regression analysis was conducted, with confession behavior as the predicted variable (0 = lie-teller, 1 = truth-teller), and condition (0 = control condition, 1 = negative moral story, 2 = positive moral story) entered as the predictor. The results showed that the model was significant, *χ*^2^ (2) = 9.65, Nagelkerke *R*^2^ = 0.14, *p* = 0.008. Experimental condition *B* = 0.98, *SE* = 0.35, Wald (1) = 7.99, *p* = 0.005, odds ratio = 2.67, with more children confessing to peeking wit the positive moral story than in the control condition: *B* = 1.83, *SE* = 0.70, Wald (1) = 6.90, *p* = 0.009, odds ratio = 6.22, 95% CI [1.59, 24.31]. Also, more children confessed to peeking in the positive story condition than in the negative moral story condition: *B* = 1.25, *SE* = 0.59, Wald (1) = 4.46, *p* = 0.035, odds ratio = 3.51, 95% CI [1.09, 11.2]. There was no significant difference in confession rates between the negative moral story condition and the control condition: *B* = 0.57, *SE* = 0.77, Wald (1) = 0.55, *p* = 0.46, odds ratio = 1.77, 95% CI [0.39, 8.06].

These results showed that positive moral story-telling made more children confess, while negative moral story-telling did not have this effect. This result is in line with findings from Lee et al. (2014). In addition, unlike previous research, this study tested Chinese preschool children, and suggests that the effect of positive moral stories on children’s honesty can hold among children from different cultures.

## 3. Study 2

Study 1 replicated previous findings that moral story-telling emphasizing truth-telling with positive consequences could promote honesty in children. However, similar to previous studies, more than sixty percent of children in Study 1 still lied after positive moral story-telling. Thus, the aim of study 2 is to examine whether we can further promote children’s honesty with a combination of external appeals and positive moral story-telling. To do so, two conditions were set up in Study 2: the *combination condition,* in which the experimenter would express that she would be happy if the children told the truth after a positive moral story and *an external appeal condition,* in which the experimenter would express that she would be happy if the children told the truth, without the positive story. We hypothesized that children in the combination condition would be more likely to confess than those in the external appeal condition and positive moral story condition alone.

### 3.1. Method

#### 3.1.1. Participants

A total of 107 children of the Han nationality were recruited from a kindergarten in an eastern city in China. Children were randomly assigned to the combination condition or the external appeal condition. Four participants were excluded because they did not understand the story, and eight participants were excluded because they did not finish the study. The valid participants in each condition were: 47 children in the combination condition (*M*_age_ = 5.76 years, *SD* = 0.25, range = 5.34 to 6.19, 32 boys) and 48 children in the external appeal condition (48 children, *M*_age_ = 5.69, *SD* = 0.33, range = 4.96 to 6.73, 30 boys). The sample size was determined by the same power analysis as Study 1. We monitored the recruitment rates and stopped collecting data when each condition had 35 children who peeked.

#### 3.1.2. Procedure

Study 2 used the same temptation resistance paradigm as Study 1 used, and the experimental procedures were the same as in Study 1, except for the condition manipulation: In the combination condition, after finishing reading the positive moral story, the experimenter said to the child “I want to ask you a question, and if you tell the truth, I will be happy”. Then, the experimenter asked, “Did you turn around and peek at the toy when I left the room?” In the external appeal condition, the experimenter did not tell any stories but still said “I want to ask you a question, and if you tell the truth, I will be happy”. Then, the experimenter asked, “Did you turn around and peek at the toy when I left the room?” The experimenter recorded the child’s responses and behavioral responses. The number of children who confessed was used as the main dependent variable in this study.

### 3.2. Results

Preliminary analyses revealed that child gender was not significantly related to lying behavior, χ^2^ (1) = 0.56, *p* = 0.45, and that age was not significantly related to lying behavior (Spearman *r* < 0.001, *p* = 0.93). Thus, gender and age were removed as factors for all further analyses. Overall, 73.7% (N = 70) of children peeked at the toy after the experimenter left the room; 74.47% (35 out of 47) of children in the combination condition peeked, and 72.92% (35 out of 48) in the external appeal condition (35 out of 48) peeked. A binary logistic regression with peeking as the predicted variable showed that there was no significant difference in peeking rate between the two conditions, χ^2^ (1) < 1, *p* = 0.86, Nagelkerke *R*^2^ < 0.01.

**Figure 2:**
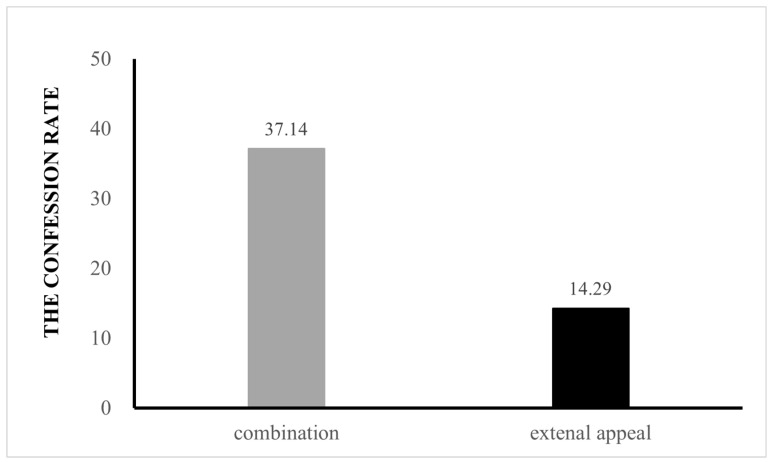
Percentage of children who confessed in Study 2.

Of the children who peeked at the toy, the confession rate in the combination condition was 37.14% (13 out of 35), and the confession rate in the external appeal condition was 14.29% (5 out of 35) (see Figure 2). To compare the confession rate between these confession rates, a binary logistic regression analysis was conducted, with confession behavior as the predicted variable (0 = lie-teller, 1 = truth-teller), and condition (0 = the external appeal condition, 1 = combination condition, 2 = positive moral story from Study 1) as the predictor. The model was significant, *χ*^2^ (1) = 4.11, Nagelkerke *R*^2^ = 0.05, *p* = 0.043. Experimental condition *B* = 0.54, *SE* = 0.27, Wald (1) = 3.93, *p* = 0.047, odds ratio = 1.72, 95% CI [1.01, 2.93], with more children confessing to peek in the combination condition than in the external appeal condition: *B* = 1.27, *SE* = 0.60, Wald (1) = 4.50, *p* = 0.034, odds ratio = 3.55, 95% CI [1.10, 11.41], and more children confessing to peeking with the positive moral story from Study 1 than in the external appeal condition: *B* = 1.22, *SE* = 0.60, Wald (1) = 4.22, *p* = 0.04, odds ratio = 3.39, 95% CI [1.06, 10.88]. However, there was no significant difference in confession behavior between the combination condition and positive moral story of Study 1: *B* = 0.04, *SE* = 0.49, Wald (1) < 0.01, *p* = 0.93, odds ratio = 1.05, 95% CI [0.40, 2.75].

## 4. Discussion

Although moral story-telling has been widely used to teach children about the virtue of honesty, the evidence suggesting its efficacy is limited. Given this, the present study sought to examine whether moral story-telling can promote Chinese preschool children’s honesty, and whether the combination of moral story-telling and external appeals would better promote children’s honesty than each technique alone. Our results showed that positive moral story-telling, rather than negative moral story-telling, promoted honesty in children aged 5–6 years old. However, the effect of the combination of the two techniques on children’s honesty was just as the same as positive moral story-telling alone.

In Study 1, we found that positive moral story-telling increased Chinese Preschool children’s honesty while negative moral story-telling did not. This result is consistent with some previous findings which show that only positive moral story-telling has a significant effect on honesty promotion (Ding et al., 2024; Lee et al., 2014; Talwar et al., 2016). We also extend previous findings by showing that positive moral stories are effective in honesty promotion in Chinese culture, since most previous research has used Western-culture samples. Social learning theory proposes that children learn social norms such as moral values by observing others. Our result supports the social learning theory by showing that children learn to behave honestly by observing story characters (Bandura, 1991). This result is also consistent with several previous studies that found that children become more honest after observing other children telling the truth (Engarhos et al., 2020; Ma et al., 2018). Study 1 also suggests that negative moral stories did not promote children’s honesty, which is likely because children may be worried about getting in trouble if they tell the truth about their rule-violation behavior.

Ding et al. (2024) found that children who were encouraged to emulate the character in the positive moral story were more likely to tell the truth than those who were not. They suggested that stories are only effective at promoting honesty among young children who are encouraged to try to be like the character in the positive moral story (Ding et al., 2024). Since we did not manipulate encouragement in this study, it is unclear whether encouragement plays an important role in promoting children’s honesty. Future studies should manipulate encouragement to investigate this issue. It should be noted that some other studies have not found that positive moral stories are effective in promoting children’s honesty (Butean et al., 2021). These studies have either used an imaginary Prince or used an animal as the moral stories’ characters. It is possible that only moral stories using actual humans are effective in promoting honesty. More studies are needed to test this hypothesis. In addition, these mixed findings may also be attributed to different cultural contexts. For example, stories from one culture may not be effective for children from another culture. Cross-cultural studies are needed to address this issue in the future.

We also sought to examine whether the combination of positive moral stories with external appeals are superior to positive moral stories alone. Inconsistent with our hypothesis, we found that the effect of the combination of the two is the same as the effect of positive moral story-telling alone. At first glance, our result is not consistent with a recent study that found that the combination of a moral story with external appeals makes children more likely to tell the truth compared to a moral story alone (Evans & Talwar, 2024). However, it should be noted that in their study, less than twenty percent of children in the moral story-telling alone condition told the truth, which suggests that a moral story alone is not effective in promoting children’s honesty. This contributes to their findings showing that the combination condition is superior to the moral story condition alone. In contrast, in the present study, around 35% of children in the positive moral story condition told the truth, which is similar to the rate in the combination condition in the study by Evans and Talwar (2024). The different confession rates for the moral story-telling alone between this study and that of Evans and Talwar (2024) may explain the different findings. Additionally, one possible reason for the null effect of the combination is that, although children were encouraged to tell the truth, they may still worry about being punished for admitting their transgressions. For example, children may experience punishment when they have told the truth in real life. In this case, children would rather keep telling lies to conceal their rule-violation, even after they learned the moral story and heard the external appeal. Further studies may examine how parents’ reactions to children’s lies in real life influence the effect of honesty promotion.

There are several limitations in this study: First, we only included one age group in this study; future studies should examine how age influences the effect of moral stories on children’s honest behavior. Several previous studies have reported that age does not influence the effect of moral stories (e.g., Lee et al., 2014; Talwar et al., 2016, 2018), while one recent study indicated that moral stories are effective only for 3- to 4-year-old children, but not for 5- to 6-year-old children (Ding et al., 2023). However, it should be noted that the null results for 5- to 6-year-old children in Ding and her colleagues’ study are due to their use of a non-pure control condition, which resulted in a high confession rate in that condition, thereby eliminating the effect of moral stories. We did not include 3-year-old children because our pilot study showed that many of them did not understand the story. This may be because the participants we tested in the pilot study were 3 years old, which is younger than those in previous studies. For example, previous studies typically included both 3- and 4-year-olds in their younger-children groups (e.g., Ding et al., 2023). Further studies could explore methods, such as presenting the stories as videos, to help young children understand the stories better. Furthermore, another recent study found that positive moral stories are effective in promoting honesty among 7- to 10-year-old school-aged children, but not among 11-year-old children (Liang et al., 2025). Future studies should include a wider range of age groups to systematically test how the effect of moral stories varies across ages. Second, in Study 2, we found that the confession rate in the combination condition was higher than that in the external appeal condition. However, it should be noted that the difference in procedure length between the two conditions may have influenced this result. Future studies should include a condition that combines external appeals with a neutral story to address this issue. Third, the current studies examined the effect of moral stories on children’s honesty immediately; future studies should examine whether there is a lasting effect of moral stories on children’s honest behavior. Fourth, the current study only examined one type of lying: lying to conceal a transgression. Further studies should consider examining whether moral stories can help to reduce other types of lying among children.

Taken together, the present study examined whether moral stories can promote 5-year-old children’s honesty in China. We found that positive moral stories rather than negative moral stories can promote children’s honesty. In addition, we found that combining moral stories with external appeals is not able to further promote children’s honesty. These findings have important implications for how to make children honest.

## Data Availability

The data will be availability upon request.
